# Hypoglycemia unawareness among insulin-treated diabetic patients in Madinah, Saudi Arabia: prevalence and risk factors

**DOI:** 10.3389/fendo.2023.1239524

**Published:** 2023-10-26

**Authors:** Amal M. Qasem Surrati, Alhanouf Ayed Alanazi, Samyah Sami Bukhari, Eman Mohammed Alfadhli

**Affiliations:** ^1^ College of Medicine, Taibah University, Madinah, Saudi Arabia; ^2^ Department of Medicine, National Guard Hospital, Medina, Saudi Arabia; ^3^ Department of Adult Endocrine, Medicine, King Fahad Hospital, Madinah, Saudi Arabia; ^4^ Department of Medicine, King Faisal Specialist Hospital and Research Center, Madinah, Saudi Arabia

**Keywords:** hypoglycemia, hypoglycemia unawareness, T1DM, T2DM, insulin, Madinah, Saudi Arabia

## Abstract

**Background:**

Hypoglycemia unawareness (HU) is associated with significant risks. Screening for impaired awareness of hypoglycemia in patients with diabetes is important to minimize those risks. There are limited data on the prevalence of HU in patients with diabetes in Saudi Arabia (KSA). In the current study, we investigated the frequency of HU and its risk factors among insulin treated diabetic patients in Madinah, KSA.

**Methods:**

A cross-sectional study was conducted in a diabetes center and four primary healthcare centers at Madinha, KSA. Patients ≥14 years old with type 1 or type 2 diabetes treated with insulin for more than a year were included. HU was assessed by Clarke’s and modified Pedersen-Bjergaard’s scores. The risk factors for HU were determined.

**Results:**

Of the 413 included patients, 60.3% were women, and 60.8% were on insulin alone. One-third of the participants had T1DM, while 68.5% had T2DM, with median ages of 25 and 56 years, diabetes durations of 10 and 15 years, and durations of insulin use of 10 and 5 years, respectively. The prevalence of HU was 25.2% by Clarke’s survey. The risk factors for HU were poor knowledge of the patient’s latest HbA1c, type of insulin, and dose of insulin. Poor medical follow-up, previous stroke, and ischemic heart disease were the other risk factors for HU. When the modified Pedersen-Bjergaard method was used, the prevalence of HU was 48.9%.

**Conclusion:**

Despite the advances in diabetes management, HU continues to be prevalent among diabetic patients on insulin, and poor diabetes knowledge is a major risk factor. Diabetes education on self-management is of utmost importance to reduce hypoglycemia and HU.

## Introduction

Hypoglycemia is a common problem in patients with diabetes receiving insulin and/or insulin secretagogue therapy. The need to ensure strict blood glucose control and attend against long-term complications of diabetes has led to this problem. Hypoglycemia is defined as a “low plasma glucose concentration that places the subject in potential harm,” and plasma glucose <70 mg/dl (3.9 mmol/L) defines hypoglycemia. A blood glucose level below 54 mg/dl (3.0 mmol/L) is considered clinically significant hypoglycemia. Severe hypoglycemia has no specific glucose threshold but instead is characterized by severe cognitive impairment demanding assistance (1).

Hypoglycemia unawareness (HU) is defined as failure to recognize a significant decline in blood glucose below normal levels, which leads to the development of neuroglycopenic symptoms before the autonomic warning symptoms (2).

In Saudi Arabia, the rate of acute complications due to hypoglycemic attacks was found to be high at 68.9% among patients with DM (3); in another local study, the prevalence of impaired awareness of hypoglycemia among patients with T1D was 62.8% (4).

Due to the standing dependence of the brain on glucose, to prevent serious consequences, an immediate counterregulatory response will be activated once blood sugar is low. Autonomic symptoms, such as palpitations, tremor, hunger, and sweating, usually occur first, which permits the recognition of “hypoglycemia awareness.” If hypoglycemia is not corrected, neuroglycopenic symptoms, such as drowsiness and cognitive impairment, develop which may progress to seizures, coma, or eventually death (2, 5).

Complications due to hypoglycemia can be serious and life-threatening, including cardiac arrhythmia, cognitive impairment, and cerebral ischemia (6).

Repeated hypoglycemic episodes contribute to the suppression of the counterregulatory hormonal and sympathetic responses, which leads to impaired awareness, hence increasing the risk for severe hypoglycemia. Hypoglycemia unawareness is associated with poor adherence to antidiabetic treatment, poor glycemic control, anxiety, depression, and poor quality of life.

The exact mechanism for the development of HU is not fully understood. Recurrent hypoglycemia causes hypoglycemia unawareness and leads to a horrendous cycle of recurrent hypoglycemia. Short-term avoidance of hypoglycemia and raising the overall mean blood glucose levels reverse hypoglycemia unawareness in many patients (5). Long duration of diabetes and long-term insulin use are negatively associated with HU. Patients with type 1 diabetes were reported to be more affected by HU than those with type 2 diabetes.

There are numerous validated self-reporting questionnaires for assessing hypoglycemia unawareness: the Gold (7), the Clarke (8), and the Pedersen-Bjergaard (9) methods. The Clarke and the Gold methods classify patients’ awareness into two categories, “aware” and “unaware,” and the Pedersen-Bjergaard method classifies awareness into three categories, “aware,” “impaired awareness,” and “severely impaired awareness.” The Clarke method encompasses eight questions evaluating the patient’s glycemic threshold and symptomatic reactions to hypoglycemia. A score of 4 or more represents HU. The Gold method asks the question “Do you know when you are getting hypoglycemia?,” which the patient answers using a seven-point scale, with 1 indicating “always aware” and 7 indicating “never aware.” A score of 4 or more indicates HU. The Pedersen-Bjergaard method (5) asks the patient the question “Can you feel when you are low?,” and the patient has to choose one response from “always,” “usually,” “sometimes,” or “never.” Patients who answer “always” are considered to have normal awareness of hypoglycemia, those who answer “never” are considered to have HU, while others are considered to have impaired awareness.

Screening individuals with diabetes for HU is important to minimize the risk of hypoglycemia by modifying glycemic targets and adjusting either insulin or insulin secretagogue therapy. Also, it was found that educating patients who are at risk of developing hypoglycemia about the types of treatment, factors causing hypoglycemia, and prevention measures is vital to reduce the health burden associated with HU (10).

There are limited data on the prevalence of HU and its risk factors in KSA. In the current study, we investigated the frequency of HU and its risk factors among insulin treated patients with diabetes in Madinah, KSA.

## Materials and methods

This was a cross-sectional study carried out in a diabetes and endocrinology center and four major primary healthcare centers in Madinah, KSA. A sample size of 400 was calculated using the Steve Thompson equation according to the estimated total number of patients with diabetes in Madinah, KSA. The inclusion criteria were T1DM or T2DM patients aged 14 years and older who had been on insulin for over 12 months. The study excluded patients with chronic liver or kidney disease, pregnant diabetics, and patients with malignancies.

The study was approved by the Research and Human Ethics Committee of King Fahad Hospital, Madinah, Saudi Arabia. Informed consent was obtained from all the participants after explaining the aim and the nature of the study.

The data were collected using a face-to-face interview questionnaire in Arabic. The form included questions that determined the patient’s demographics, disease characteristics, management, complications, and latest HbA1c. Awareness of hypoglycemia was assessed by two methods: Clarke’s (7) and modified Pedersen-Bjergaard’s (9) surveys. In the modified Pedersen-Bjergaard method, we asked the patient the question “Can you feel when you are low?,” and the patient had to select one response from three responses instead of four—”always,” “sometimes,” or “never”—which represent normal, impaired, and severely impaired awareness, respectively.

Participants were divided into two groups: aware and unaware according to Clarke’s survey, and comparisons were made between the two groups.

The data analysis was performed using Statistical Packages for Social Sciences (SPSS) version 21. Continuous variables were expressed as the mean ± standard deviation (SD) or median [interquartile range (IR)] as appropriate, and categorical variables were expressed as numbers (percentages). An independent *t*-test was used to test for differences in the continuous variables, and a chi-square analysis was used to test for differences in the categorical variables. *P <*0.05 was used as the cutoff value for significance.

## Results

Of the 413 patients included in the study, 249 (60.3%) were women, 251 (60.8%) were treated with insulin only, while 162 (39.2%) were treated with both insulin and oral hypoglycemic agents. One hundred thirty patients (31.5%) had T1DM, and 283 (68.5%) had T2DM, with median (IR) ages of 25 (21–42.25) and 56 (50–62) years, median (IR) diabetes durations of 10.0 (7–17) and 15.0 (9–20) years, and median lengths of insulin use of 10.0 and 5.0 years, respectively. The clinical characteristics of the participants are shown in **Table 1**.

**Table 1:** Demographic data of 413 patients with diabetes.

Study variables	Total *N* = 413	T1DM *N* = 130 (31.5%)	T2DM *N* = 283 (68.5%)
Age (years)*	52	25	56
Male/female	39.7/60.3	36.9/63.1	41.0/59.0
Nationality (Saudi/non-Saudi)	92.5/7.5	91.5/8.5	92.9/7.1
Duration of DM (years)*	14.0	10.0	15.0
Duration of insulin use (years)*	8.5	10.0	5.0
HbA1c (%)*	8.6	8.59	8.64
HbA1c <7	12.9	15.5	11.5
Managed by insulin only/insulin and OHA	60.8/39.2	95.4/4.6	44.9/55.1
Patient’s knowledge of insulin dosage	79.4	78.5	79.9
Patient’s knowledge of insulin type	44.3	63.1	35.7
Patient’s knowledge of the latest HbA1c level	58.6	64.6	55.8
Regular medical follow-up	81.1	78.5	82.3
Daily SMBG	47.0	46.9	47
Prevalence of HU as per Clarke’s survey	25.2	25.4	25.2
Prevalence of HU as per modified Pedersen-Bjergaard’s survey	48.9	45.4	50.5

The data are presented in percentages (%) and those indicated with * are in medians.

DM, diabetes mellitus; T1DM, type 1 diabetes mellitus; T2DM, type 2 diabetes mellitus; HbA1c, glycated hemoglobin; OHA, other hypoglycemic agents; SMBG, self-monitoring of blood glucose.

When Clarke’s survey was used, HU was documented in 104 patients, reflecting an overall prevalence of 25.2% with no differences between T1DM and T2DM. The risk factors for HU were poor knowledge of the patient’s latest HbA1c level (*P* = 0.002) and of the type and dose of insulin (*P* = 0.001). Poor medical follow-up (*P* = 0.003), previous stroke (*P* = 0.003), and ischemic heart disease (*P* = 0.048) were associated with increased risk for HU. Patients with HU were using significantly lower doses of daily insulin than the aware group (*P* = 0.030). Hypoglycemia unawareness was not dependent on age, gender, duration of diabetes, duration of insulin therapy, HbA1c, frequency of blood glucose monitoring, or microvascular complications of diabetes. In addition, we did not find differences in HU between patients receiving insulin alone and those receiving both insulin and other hypoglycemic agents (**Table 2**).

**Table 2:** Risk factors for hypoglycemia unawareness as assessed by Clarke’s method.

Variables	Aware group (*n* = 299)	Unaware group (*n* = 104)	*P*-value
Age (years)*	47.7	49.6	0.303
Male	38.5	43.3	0.418
Female	61.5	56.7	0.418
Duration of DM (years)*	14.9–15.0	13.6	0.183
Type 1 DM	31.4	31.7	1.000
Type 2 DM	68.6	68.3	1.000
HbA1c*	8.5	8.9	0.158
HbA1c <7	14.4	6.5	0.220
Duration of insulin use (years)*	8.61	7.9	0.369
Insulin dose (total units/day)*	56.2	46.9	0.030
Managed by insulin only	61.2	40.4	0.817
Managed by insulin and OHA	38.8	59.6	0.817
Lack of knowledge of insulin type	50.8	70.2	0.001
Lack of knowledge of insulin dosage	16.5	32.7	0.001
Lack of knowledge of the latest HbA1c level	36.9	54.8	0.002
Lack of medical follow-up	15.5	28.8	0.003
Lack of daily SMBG	52.4	54.8	0.380
Previous stroke	9.7	19.2	0.003
Ischemic heart disease	14.6	25.0	0.048
Diabetic neuropathy	35.9	36.5	0.384
Diabetic retinopathy	46.0	45.2	0.409
Diabetic nephropathy	12.6	18.3	0.357

The data are presented in percentages (%) and those indicated with * are in means.

DM, diabetes mellitus; T1DM, type 1 diabetes mellitus; T2DM, type 2 diabetes mellitus; OHA, other hypoglycemic agents; HbA1c, glycated hemoglobin; SMBG, self-monitoring of blood glucose.

When HU was evaluated with the modified Pedersen-Bjergaard method, the prevalence was 48.9% (41.9% had impaired awareness and 7% had no awareness), with T2DM patients being affected at a slightly greater rate than those with T1DM (*P* = 0.038), 50.5% and 45.4%, respectively.

## Discussion

In the current study, the prevalence of HU as assessed by the Clarke questionnaire score was 25.2% with no differences between T1DM and T2DM. This result is consistent with the results of many previous studies (11–13) but higher than reported in Jordan, where the prevalence of HU in patients with insulin-treated T2DM was 17.01% as determined by Clarke’s method (14).

When HU was evaluated by the modified Pedersen-Bjergaard method, a much higher prevalence of HU was observed (48.9%, almost double), with T2DM patients being affected by a more severe impairment of awareness than T1DM (*P* = 0.038). This result from the current study is consistent with previous studies that used the Pedersen-Bjergaard method (9), in which the prevalence of HU was between 52% and 63% in T1DM and 52% and 56.5% in T2DM patients (15–19). The Pedersen-Bjergaard method tends to overestimate the prevalence of HU as documented previously by Geddes et al. (12) That study evaluated the concordance between the three methods for assessing awareness of hypoglycemia—the Gold (7), the Clarke (8), and the Pedersen-Bjergaard (9) methods—in patients with T1DM and found a strong correlation between the Clarke and Gold methods but poor correlation between the Pedersen-Bjergaard method and the other two methods. The prevalence of HU was 24%, 26%, and 62.5%, as observed by the Gold, Clarke, and Pedersen-Bjergaard methods, respectively (7). A higher prevalence of HU was reported in T2DM patients from Turkey (83.4%) (20), while a lower prevalence of HU was reported from Brazil (20% in T1DM and 35% in T2DM) (21).

The factors reported to affect HU are not consistent among different studies, and some factors that were demonstrated to increase the risk for HU in some studies were not confirmed in others. However, long diabetes duration and strict blood sugar control are the most commonly reported factors that raise the risk of HU (12). Nevertheless, in the present study, patients with HU had disease durations and HbA1c levels similar to those of aware subjects, findings that were also documented in other studies (14). However, some studies found that patients with HU have higher HbA1c values (20). Relaxing the glycemic target in patients with HU could explain the higher HbA1c values in those patients. In the current study, patients with HU were using lower daily doses of insulin than the aware group (*P* = 0.030); this difference in dose might be a consequence and not the cause of reduced awareness.

Interestingly, we found that poor diabetes knowledge, such as poor knowledge of a patient’s insulin type or dosage or latest HbA1c level, and also poor medical follow-up are risk factors for hypoglycemia unawareness. Similarly, Murata et al. found that inadequate knowledge of diabetes is a risk factor for HU in type 2 diabetes (22). Alanazi et al. also found that poor awareness of hypoglycemic attacks was observed among 62.6% of the participants (23). Another local study found that 92.2% of the participants had a poor level of knowledge (24).

Diabetes education is a crucial key in diabetes management and should be a continuous process to improve blood glucose control, avoid hypoglycemia, and reduce diabetic complications.

In the current study, we found that macrovascular complications of diabetes, specifically previous stroke and ischemic heart disease, are associated with increased risk for HU, whereas diabetic neuropathy and other microvascular complications of diabetes are not. Contrary to these results, Murata et al. found that stroke had no effect on hypoglycemia awareness, and intriguingly, the presence of microvascular complications of diabetes was associated with less risk for HU (22).

The findings from previous studies revealed that a significant number of patients with T1DM and T2DM were reluctant to discuss their hypoglycemia with their healthcare provider (HCP). There could be many reasons for such a dangerous attitude, including implications for employment, fear of losing driving privileges, or concerns that it discloses poor glycemic control to the HCP (15–18). In view of these findings along with the great risk of hypoglycemia associated with HU, regular screening for HU is a crucial element of diabetes care. For insulin-treated patients with HU, they are advised to raise their glycemic targets to strictly avoid hypoglycemia for at least several weeks in order to partially reverse hypoglycemia unawareness and reduce the risk of future episodes.

Our study is limited by the use of a non-laboratory method to assess HU and the reliance on patients’ self-reported questionnaires that are subject to bias. However, we used two validated questionnaires commonly used in other studies for assessing HU (7, 9). A further limitation of our study is that it was conducted only in one area of Saudi Arabia, so it may not be applicable to other Saudi populations. Despite these limitations, our findings provide valuable insights into HU in KSA. In addition, this study is among the few studies that investigated HU in Saudi Arabian insulin-treated diabetic patients. The study also provides valuable information on the association between HU and diabetes education. Further research is needed to confirm and extend our results. In addition, interventions to improve HU should also be explored.

## Conclusion

Despite the advances in insulin formulations and technologies used to control diabetes, HU continues to affect a significant proportion of patients with diabetes on insulin. Poor diabetes knowledge is a major risk factor for HU. Structured education for effective self-management of diabetes and screening for impaired awareness of hypoglycemia are of utmost importance to improve glycemic control and reduce the risk of hypoglycemia.

## Data availability statement

The raw data supporting the conclusions of this article will be made available by the authors, without undue reservation.

## Author contributions

EA conceptualized the idea of the research, and wrote the manuscript. AS was responsible for the literature search and provided research materials. SB collected and organized the data and references and provided logistic support. AA was responsible for data collection. All authors contributed to the article and approved the submitted version.
